# Interactions of Phosphate Metabolism With Serious Injury, Including Burns

**DOI:** 10.1002/jbm4.10011

**Published:** 2017-07-05

**Authors:** Craig Porter, Linda E Sousse, Ryan Irick, Eric Schryver, Gordon L Klein

**Affiliations:** ^1^ Department of Surgery University of Texas Medical Branch at Galveston Galveston TX USA; ^2^ Shriners Burns Hospital University of Texas Medical Branch at Galveston Galveston TX USA; ^3^ Department of Orthopaedic Surgery and Rehabilitation University of Texas Medical Branch at Galveston Galveston TX USA

**Keywords:** PHOSPHORUS, TRAUMATIC BRAIN INJURY, BURNS, SPINAL CORD INJURY, PARATHYROID HORMONE

## Abstract

Approximately 85% of the body's phosphate pool resides within the skeleton. The remaining 15% is stored as high‐energy phosphates or in its free form, where it acts as a substrate for adenosine triphosphate (ATP) production. Accordingly, phosphate plays a crucial role in energy metabolism. Trauma and critical illness result in a hypermetabolic state in which energy expenditure increases. The impact of trauma and critical illness on the body's phosphate stores and phosphate‐dependent metabolic reactions is poorly understood. We had previously observed that after severe burn trauma, increased energy expenditure is temporally related to a marked reduction in serum concentrations of both parathyroid hormone and fibroblast growth factor 23, both of which have phosphaturic effects. The aim of this article is to describe as far as is known the similarities and differences in phosphate metabolism in different types of injury and to infer what these differences tell us about possible signaling pathways that may link increased phosphate utilization and phosphate retention. © 2017 The Authors. *JBMR Plus* is published by Wiley Periodicals, Inc. on behalf of the American Society for Bone and Mineral Research.

## Introduction

Phosphate metabolism in major trauma, such as burns, multiple fractures, spinal cord injury, or traumatic brain injury, is little understood. It is not even certain that it is similar from one type of injury to another, and the relationship of phosphate metabolism to differing severity of each type of injury also remains unknown. The purpose of this review is to draw on our experience with severe burns to identify aspects of phosphate metabolism that may not have been previously considered and that may be present in a variety of traumatic insults. Traumatic injuries, including burns, have no specifically evolved adaptive responses, and the body's response to them draws on nonspecific adaptive mechanisms. Therefore, identification of the mechanisms controlling phosphate metabolism during injury may provide insight as to how the body regulates energy metabolism during stress.

We will first discuss what is known about phosphate metabolism during severe burns and will then attempt to identify features of phosphate metabolism that may be similar in other types of traumatic injury. This approach may help us identify common responses to serious injury.

## Burns

**Figure 1:**
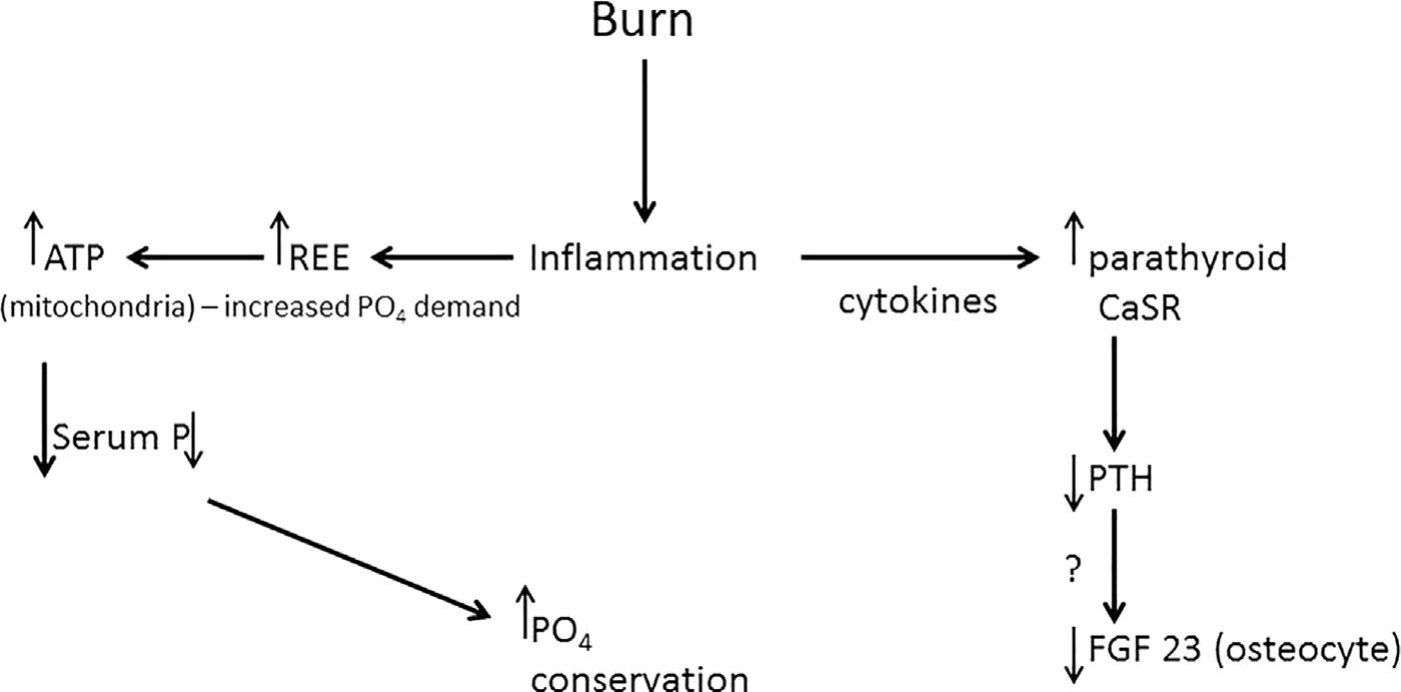
A schematic diagram of the possible signaling pathway or pathways linking increased intracellular phosphate utilization and phosphate retention.

Fig. 1 outlines the generalized loop that describes how the body may meet increased phosphate demands after severe burns. Destruction of the skin as a barrier to the entry of microorganisms results in wound infections and a consequent systemic inflammatory response. Concomitantly, an increase in energy expenditure results in an increase in production of adenosine triphosphate (ATP) by mitochondria. Although all aspects of how the body meets the demand for increased phosphate utilization are not known, we have identified several areas of interest, each of which will be discussed. These are as follows: 1) the impact of increased phosphate demand for ATP production; 2) effect on circulating and tissue phosphorus concentration; 3) renal phosphate retention and excretion; 4) importance of glucocorticoids; 5) interactions with other metabolic pathways; 6) possible differences between pediatric and adult patients.

## Increased Phosphate Demand for ATP Production

Phosphorus is the second most abundant mineral in the body. After ingestion, it is absorbed in the small intestine, enhanced by 1,25‐dihydroxyvitamin D, and its excretion is controlled exclusively by the kidney. Phosphorus accounts for approximately 1.1% of total body mass. Thus, an 80 kg individual has a total body phosphorus content of approximately 880 g, about 85% of which can be found in the skeleton as calcium phosphate. A significant portion of the body's nonskeletal phosphate stores resides within skeletal muscle either as free inorganic phosphorus (Pi) or bound within high‐energy phosphate molecules (HEP), such as ATP, adenosine diphosphate (ADP), adenosine monophosphate (AMP), or phosphocreatine (PCr). Skeletal muscle has a Pi content of approximately 45 mmol/kg dry weight. Moreover, at rest, ATP (25 mmol/kg dry weight), ADP (3.3 mmol/kg dry weight), AMP (0.1 mmol/kg dry weight), and PCr (80 mmol/kg dry weight) collectively contain about 70 mmol/kg dry weight of Pi. Using these values, we can estimate that skeletal muscle has a Pi concentration of 2.7 g/kg. For an 80 kg individual with 32 kg of muscle mass (40% of total mass), approximately 85 g of Pi resides in skeletal muscle. This represents about 10% of the body's total phosphorus stores. Skeletal muscle plays important roles in both the whole body protein metabolism and locomotion, both of which functions are highly dependent on ATP. Therefore, muscle is endowed with numerous cristae dense mitochondria to support fuel oxidation and oxidative phosphorylation. As a principal substrate of ATP synthetase, Pi clearly plays a critical role in supporting ATP production. Furthermore, HEPs such as ATP and PCr are stored in skeletal muscle in limited amounts to provide a temporal energy supply to power metabolic processes and muscle contraction when energy demand outpaces mitochondrial ATP production rates.

## The Impact of Burn Trauma on Skeletal Muscle Phosphate Metabolism

Because trauma and burns deplete muscle Pi levels, the question arises, do reduced Pi levels hinder energy metabolism in muscle? Hypermetabolism is a hallmark of the stress response to burns, and skeletal muscle ATP turnover is known to be elevated after burn trauma, owing largely to greater protein synthesis and breakdown rates. ATP is depleted in muscle after trauma,^1^ including patients with burn trauma.^2^ Indeed, in addition to Pi, HEPs are also reduced in the skeletal muscle of burn patients. Loven and colleagues^2^ reported a 25% decrease in ATP and a 15% reduction in PCr in muscle of trauma patients including those with severe burn. The concurrent reduction in muscle Pi and HEPs in muscle in response to burns suggests that Pi depletion may be limiting to ATP production because Pi is a substrate for ATP synthase. However, muscle ADP levels are also depleted in trauma and burn patients, whereas muscle AMP levels are increased in these patients.^3^ The absence of a buildup of ADP suggests that reduced Pi may not be limiting to oxidative phosphorylation. Moreover, animal^4^, ^5^ and human^1^, ^2^ data suggest that there is increased glycolytic flux in muscle after severe trauma, and PCr depletion may reflect increased ATP turnover rates and a shift in cellular fuel metabolism away from oxidative phosphorylation and toward greater substrate level phosphorylation, meaning reduced ATP content in muscle of trauma and burn patients may be independent of Pi levels.

Overall, there is a paucity of data regarding the impact of burn trauma on muscle phosphate levels. Although a few studies have reported that Pi and HEP are depleted in skeletal muscle in burns and trauma and the ATP turnover is increased, whether reduced Pi levels are limiting to ATP production rates remains unknown. Future studies are needed to address these questions. Further, in addition to lower Pi levels, reduced magnesium (Mg) content may be an alternative explanation for reduced ATP levels in skeletal muscle in burns and trauma. Despite being constantly produced by mitochondria, ATP is stored at a level of around 25 mmol/kg dry tissue in muscle, meaning muscle has a readily available store of ATP. In vivo, ATP is chelated to Mg, which suggests that reduced muscle Mg levels may underlie lower ATP content in skeletal muscle of trauma patients. However, this assertion remains speculative at present.

## Effect of Increased Phosphate Demand on Circulating Concentrations of Phosphorus

In an analysis of de‐identified data from 181 pediatric patients with total body surface area burns of ≤20%, there is an acute hypophosphatemia (<3.5 mg/dL)^6^ with onset a mean of 2.2 ± 1.4 (SD) days from admission in 49% of the patients. The mean value was 2.84 ± 0.51 mg/dL (*n* = 399), with a range of 0.8 to 3.4 mg/dL. Serum phosphorus recovered to normal after a mean of 4.7 ± 5 days, range 1 to 15 days. Total monitoring time was 7.8 ± 7.1 days, range 1 to 33 from onset of burn injury. These patients would have received a potassium phosphate supplement orally for serum phosphorus <3.2 mg/dL or oral NeutraPhos^R^, 250 mg phosphorus per packet, for serum phosphorus concentrations from 3.2 to 3.5 mg/dL.

One possible source of additional phosphate is bone resorption, which occurs starting within the first 24 hours post‐burn in a sheep model of 40% body surface area burn injury.^7^ However, this source is limited given that bone turnover decreases dramatically by 2 weeks post‐burn, resulting in a hypodynamic or adynamic state.^(8)^ When mean values of serum phosphorus are taken for the entire acute hospitalization, the values fall within the normal range, 4.8 ± 2.8 mg/dL, and do not appear to differ from the reported pattern in more severely burned patients.^9^, ^10^ Furthermore, given the acute low levels of serum phosphorus concentration, it would not appear as if the amount of bone resorption liberates sufficient phosphate to satisfy the increased demand for phosphorus to buffer serum levels. Therefore, the rise of serum phosphorus to normal concentrations in serum must be aided by other mechanisms.

## Increased Phosphate Retention

Phosphorus loss in the blood and urine has not been systematically examined in pediatric burn patients. Urinary phosphate excretion has rarely been measured in burn patients, especially using the tubular maximum phosphate reabsorption calculation, or TmP/GFR. This value corresponds to the serum concentration of phosphate below which all filtered phosphate would be reabsorbed by the kidney. This has been done once as a modified Ellsworth‐Howard test, which relies on a 10‐minute infusion of 3 to 5 units/kg human parathyroid hormone (PTH) 1‐34 and the monitoring of urinary cyclic AMP and TmP/GFR. The fall in TmP/GFR was a mean of 13% compared with an expected mean fall of 28%,^(11)^ indicating that there is some resistance to both the expected rise in urinary cyclic AMP and the phosphaturic effects of PTH at the level of the kidney. The mechanism(s) that initiate the renal resistance to the effects of PTH is (are) not clear. Renal phosphate transporters have not been studied in burn injury and could possibly be affected by the catabolic changes after burn injury, but this is speculation at the present time. Hypoparathyroidism, which is also found in pediatric burn patients,^11^ will be discussed in the next section and could also play a role.

Further studies of proteins produced by the osteocyte, fibroblast growth factor 23 (FGF 23) and sclerostin, in patients who had entered a randomized, prospective, double‐blind trial of a single dose of the bisphosphonate pamidronate within the first 10 days after burn injury^12^, ^13^ revealed an interesting pattern. In placebo controls, serum concentrations of both FGF23 and sclerostin were undetectable.^14^ However, when samples were taken over time, the placebo controls demonstrated persistent low‐serum sclerostin concentrations, whereas those who had received pamidronate experienced a progressive rise in sclerostin concentration. Although it is not clear at what serum concentration sclerostin begins to impair osteoblastogenesis as a result of interference with the Wnt signaling pathway, the rise in sclerostin is consistent with an increase in bone mass and possibly an increase in osteocytes. In contrast to the rise in sclerostin, serum FGF23 remained undetectable in pediatric patients given pamidronate as well as those who served as placebo controls.^14^ Therefore, something was suppressing FGF23 in both groups. This something is possibly PTH suppression^11^ inasmuch as PTH is proposed to affect FGF23 concentration in serum and calvarium in a mouse model of hyperparathyroidism,^15^ although the intact FGF23 molecule is rapidly degraded, as shown in a more recent mouse model.^16^ Others, however, have concluded that it is not PTH but 1,25‐dihydroxyvitamin D that stimulates FGF23 production,^17^ whereas still others feel that PTH suppresses FGF23 production, at least acutely.^18^ It is not certain if any of these models are appropriate for the study of pediatric burns. Regardless of mechanism, the end result is that the two chief phosphaturic hormones in the body are suppressed in children after severe burn injury.

## Possible Role of Glucocorticoids

Do glucocorticoids have a role in the fall in serum phosphorus concentration with burn injury? The large quantities of endogenous glucocorticoids produced in response to burn injury^8^, ^19^ could potentially reduce phosphate absorption or increase phosphate excretion as both have been described in animal studies.^20^, ^21^ A reduction in phosphate absorption by the intestine after glucocorticoid infusion has been described in pigs.^20^ Although the mechanism may be multifactorial,^20^ a glucocorticoid‐mediated reduction in renal brush border phosphate uptake may result in altered regulation of the sodium phosphate co‐transporter.^21^ Additionally, the phosphate‐regulating gene with homologies to endopeptidases on the X chromosome (PHEX), the malfunction of which can lead to X‐linked hypophosphatemic rickets, contains a glucocorticoid response element, and animal studies have shown that PHEX is upregulated in glucocorticoid‐treated suckling mice.^22^

Glucocorticoid‐induced gluconeogenesis doesn't appear to affect renal phosphate handling as studies by Yanagawa and colleagues^23^ using isolated rabbit proximal tubules failed to demonstrate a relationship between changes in rates of gluconeogenesis and phosphate fluxes. Additionally, there has been a case report of pseudoaldosteronism in association with elevated urine free cortisol and hypophosphatemia.^24^ However, the increase in endogenous glucocorticoid production was attributed to the stress from the development of pneumonia after the elevation of aldosterone and the hypophosphatemia and elevated urine free cortisol were considered to be independent of the rise in aldosterone. Although glucocorticoids may play a role in the transient fall in serum phosphorus concentration, the return to normal of serum phosphorus concentration suggests that other factors are of greater significance in maintaining normal serum phosphorus concentrations.

As will be discussed, inflammatory cytokines are capable of upregulating the parathyroid calcium‐sensing receptor in children, thus reducing PTH secretion and consequently the phosphaturic capacity of the body. This has not been observed in adult burn patients. Aside from the potential effects of glucocorticoids on phosphate absorption and excretion, these hormones, as well as inflammation and sepsis, all contribute to oxidative stress. As an accommodation to this phenomenon, the Forkhead Box O (FOXO) transcription factors stimulate two muscle‐specific ubiquitin ligases, muscle ringfinger protein‐1 (MuRF‐1) and atrogin‐1 muscle F box, resulting in ubiquitination, which targets sarcomeric proteins for degradation.^25^, ^26^ Additionally, Sriram and colleagues^27^ have shown that part of this cachexia pathway could be stimulated by myostatin, which produces reactive oxygen species in myoblasts leading to damage and subsequent breakdown of muscle proteins. How these two pathways interrelate is not clear at present. Also to date, it is unclear whether the effects of oxidative stress on FOXO transcription factors represent a final common pathway or whether the effects of inflammation and sepsis are mediated by glucocorticoids.

There is a possibility that higher serum phosphorus concentrations may be associated with skeletal muscle wasting inasmuch as one study has associated higher serum levels of phosphorus in patients with heart failure who were more catabolic.^28^ We are unaware of studies such as this in burns or trauma.

## Interactions with Other Metabolic Pathways

### The calcium‐sensing receptor

These studies are also limited, but we do know that in pediatric burns the cytokines produced by the systemic inflammatory response, interleukins (IL)‐1β and IL‐6, can both upregulate the parathyroid calcium‐sensing receptor (CaSR),^29^, ^30^, ^31^ resulting in a reduced set point for circulating calcium suppression of PTH secretion and creating a hypocalcemic hypoparathyroid state.^11^ In our sheep model of burn injury, we observed a 50% upregulation of the parathyroid CaSR by 48 hours post‐burn.^32^ The creation of a hypoparathyroid state then leads to a possible failure to stimulate osteocyte production of FGF23 or, at the very least, a diminution of phosphaturic capability of the body.

### Magnesium depletion

Magnesium depletion is produced by the acute fluid resuscitation of burned children with Ringer's Lactate Solution, which lacks magnesium.^11^ Although we initially produced a state of magnesium repletion in approximately half our pediatric burn patients by treating them intensively with magnesium intravenously, we based our conclusion on the magnesium loading test, which indicates that an increase in magnesium excretion in the urine is a measure of adequacy of magnesium stores.^11^ However, upregulation of the CaSR also increases the quantity of magnesuria,^33^ therefore confounding the results of a magnesium loading test and obscuring ongoing magnesium depletion, which will result in renal resistance to the effects of PTH. Therefore, Mg depletion does in fact occur after pediatric burn injury, and this occurrence may well affect the availability of ATP for metabolic reactions in muscle as was discussed earlier.

### Vitamin D deficiency

Although 1,25‐dihydroxyvitamin D is capable of enhancing intestinal absorption of both calcium and phosphate, the evaluation of vitamin D status is difficult to assess immediately post‐burn because of the extravasation of plasma binding proteins, including Vitamin D Binding Protein and albumin.^34^ Although albumin levels in the serum can return to normal as early as 6 months post‐burn,^35^ it is not entirely clear as to when Vitamin D Binding Protein returns to normal. What we do know is that 1,25‐dihydroxyvitamin D levels are normal at 2 years post‐burn and start to fall at around 7 years post‐burn.^34^ This speaks to a gradual development of vitamin D deficiency and not one that occurs early on. We would thus argue that acute deficiency of 1,25‐dihydroxyvitamin D would not play a role in post‐burn phosphate metabolism, although maintenance of normal circulating concentrations of 1,25‐dihydroxyvitamin D immediately post‐burn may maximize the intestinal absorption of phosphate during the period of increased demand. Later on, 1,25‐dihydroxyvitamin D deficiency may affect the ability of the parathyroid CaSR to respond to different concentrations of magnesium, possibly contributing to magnesium depletion.^36^ Moreover, although vitamin D deficiency pre‐burn could potentially place burn patients at higher risk for phosphate deficiency, in practice there is no way to know this, and once the burn occurs, it is not possible to evaluate 25‐hydroxyvitamin D status as stated in the text.

## Possible Differences in Phosphate Conservation Between Pediatric and Adult Burn Patients

Evidence is emerging that the calcemic response to burn injury is different between children and adults. Our studies^11^ and those of Gottschlich and colleagues^37^ have indicated that pediatric patients develop hypocalcemic hypoparathyroidism after a burn injury, whereas studies of adults indicate that patients severely burned are either normocalcemic or mildly hypercalcemic and are euparathyroid or mildly hyperparathyroid.^38^, ^39^ These data suggest that whereas pediatric burn patients experience an upregulation of the parathyroid CaSR as described above, adult burn patients do not. Additionally, it must be borne in mind that phosphate demands are higher in children than in adults as reflected in the lower limits of adult normal values. In children, this is 3.5 mg/dL, whereas in adults, it is 2.0 mg/dL. Although little data are available in adults with burns on FGF23 and sclerostin, one study does report elevated serum concentration of FGF23,^39^ indicating ongoing phosphaturia. While it is not known whether mitochondrial production of ATP is increased in adults, if phosphate is not conserved in adults and mitochondrial ATP production is elevated, there is a possibility that adults could become phosphate depleted more rapidly than children. This phosphate depletion could lead to the increased morbidity compared with pediatric burns patients as has previously been documented.^40^

## Effects of Other Forms of Injuries on Phosphate Metabolism

Phosphate metabolism in other forms of trauma have not been well studied, although there are some interesting reports on phosphate in both spinal cord injury and in traumatic brain injury. Hypophosphatemia is reported in both types of injury.

In 2009, a study of 50 multiple trauma patients, 25 of them with traumatic brain injury (TBI), had serum phosphorus concentrations determined. Although overall there was no difference between those with traumatic brain injury and those without, on day 3 of hospitalization, patients with TBI had a lower serum phosphorus concentration, 2.5 ± 0.5 mg/dL versus those without TBI, 2.9 ± 0.7 mg/dL, *p* < 0.05. Also of note, in this same study,^41^ mean net phosphorus intake was greater in trauma patients with TBI than in those without, *p* < 0.001, implying ongoing phosphate loss in the population of trauma patients with TBI.

In 2000, a study by Poldermann and colleagues^42^ using a similar design showed on admission that serum phosphorus in trauma patients with TBI was 0.56 ± 0.17 mmol/L (range 0.20 to 0.92, *n* = 18) compared with 1.11 ± 0.15 mmol/L (range 0.88 to 1.44, *p* < 0.01 in those without TBI, *n *= 19, *p* < 0.01). Furthermore, in the trauma patients with TBI, 11 of the 18 patients had a serum phosphorus concentration below 0.6 mmol, whereas none of the patients in the non‐TBI trauma group experienced phosphorus levels this low (*p* < 0.01). A potential confounding factor in brain‐injured trauma patients is that they are subject to polyuria, including cerebral salt loss, mannitol diuresis, and inappropriate ADH secretion, in which the hypophosphatemia may be dilutional in the latter condition. In a study of an overall population of critically ill patients, Suzuki and colleagues found that the incidence of severe hypophosphatemia, serum phosphorus concentration below 0.6 mmol/L, was only 18 of 106 patients, or 17%.^43^ In contrast, in a 1985 study of 5 head trauma patients, Gadasseux and colleagues^44^ found that all 5 developed hypophosphatemia to less than 1 mg/dL within the first 24 hours after injury before recovering 48 hours later.

Phosphate excretion has not been extensively studied in trauma victims. As previously mentioned, both polyuria and the syndrome of inappropriate ADH secretion could contribute to hypophosphatemia. French and Bellomo in 2004^45^ studied phosphate excretion in a patient with spinal cord injury and found an elevation in the TmP/GFR up to 5 mmol/L after an infusion of 14.5 mmol phosphate ions. This result suggests phosphate retention as the normal TmP/GFR is 0.8 to 1.35 mmol/L. This phosphate retention is consistent with a pattern of hypoparathyroidism that was reported by Mechanick and colleagues in 1997,^46^ in which patients with spinal cord injury had a suppression of PTH compared with those with TBI, 13.4 ± 1.1 pg/mL compared with 27.3 ± 3.9 pg/mL. In the study of Gadasseux mentioned above,^44^ the 5 patients with TBI who all had serum phosphorus concentrations consistently below 2 mg/dL all had persistent elevations of urinary phosphate excretion in excess of 100 mg/d.

Magnesium (Mg) deficiency may also play a role in phosphate depletion. In the study by Poldermann and colleagues in 2000^42^ previously discussed, the 18 patients with TBI were compared against the 19 trauma patients with multiple fractures but no TBI. In the trauma patients with TBI, 12 of 18 (67%) had Mg concentration in the serum below 0.70 mmol/L versus only 2 of 19 (10.5%) in the multiple fracture group (*p* < 0.01). Interestingly, when head trauma is not incurred, there are no differences in magnesium concentration in serum between pediatric trauma patients with fracture and a comparable normal pediatric population as reported by Chan and colleagues in 1984.^47^

## Possible Interventions

Although increased muscle protein turnover increases the metabolic demand for phosphate and possibly magnesium, one potential way of meeting that demand would be to reduce the turnover of muscle protein. A potential example of this would be in severe burns, where there is net catabolism of skeletal muscle. However, a recent retrospective study of pediatric patients who participated in a randomized, double‐blind, controlled study of single‐dose pamidronate administration within the first 10 days after burn injury indicated that there was a reduction in both fractional synthetic and fractional breakdown rates of muscle protein,^12^, ^13^ which would likely result in a reduced requirement for HEPs. Other anabolic agents, such as recombinant human growth hormone^48^ or oxandrolone,^49^ may also accomplish the same thing.

## Conclusions

Thus, from this limited examination of phosphate metabolism after traumatic injury, we see from the study of burns that pediatric patients suffer significant but transient hypophosphatemia with a more lasting hypomagnesemia, the latter likely because of upregulation of the parathyroid CaSR and fluid resuscitation with Mg‐free Ringer's Lactate solution. There is tissue depletion of phosphate along with the hypophosphatemia, and at the same time, there is an increase in ATP turnover because of the increased demands for use in protein synthesis and breakdown. Of note is that during the increase in intracellular demand for phosphate and some overall reduction in tissue phosphate, there is a marked reduction in the two hormones that facilitate phosphate excretion, PTH and FGF23.

When other forms of trauma are examined, limited reports on spinal cord injury suggest that some of the features of burns are found as well, especially the fall in PTH and the single report of urinary phosphate retention as denoted by the high TmP/GFR. This is consistent with the failure of infused PTH to cause the expected drop in TmP/GFR after severe pediatric burns. It is unclear if Mg depletion is similar in patients with burn and spinal cord injuries as Mg deficiency has not to date been reported in spinal cord injuries. However, low PTH concentrations in both burns and spinal cord injuries may suggest that patients with spinal cord injuries have upregulation of the parathyroid CaSR. This has yet to be examined.

What requires an explanation is the temporal relationship between the increase in intracellular ATP utilization, the drop in muscle inorganic phosphate and high‐energy phosphate (HEP) content, and the shutdown of both PTH and FGF23 in pediatric burn injury along with the failure of a test infusion of PTH to lower TmP/GFR, thus increasing phosphate excretion. This relationship would appear to be an attempt to conserve phosphate by the body as shown in Fig. 1. Although we do not have enough information to address this question at the present time, perhaps the answer may lie in the study of patients with traumatic brain injury. In those patients, hypophosphatemia may be more severe than in the other forms of trauma and yet these patients appear to continue to excrete large quantities of phosphate and have normal serum concentrations of PTH. Therefore, it is possible that the drop in PTH and FGF23 may be critical to the preservation of adequate amounts of phosphate to support the increased intracellular phosphate utilization, at least in muscle, and that trauma to the brain may disrupt the signaling pathway or pathways that lead to the reduction in phosphate excretion.

## Disclosures

All authors state that they have no conflicts of interest.
